# Measured Postoperative D_L,CO_ and FEV_1_ Frequently Exceed Segment-Count Predictions After Lung Cancer Surgery: A Retrospective Cohort Study

**DOI:** 10.3390/cancers18213576

**Published:** 2026-11-06

**Authors:** Helene Louise Hartmeyer, Cæcilie Kaas Bang, Gitte Alstrup, Morten Bendixen, Uffe Bodtger

**Affiliations:** 1 Respiratory Research Unit PLUZ, Department of Internal and Respiratory Medicine, Zealand University Hospital, 4700 Næstved, Denmark; helene.hartmeyer@live.dk (H.L.H.); cabn@regsj.dk (C.K.B.);; 2 Department of Thoracic Surgery, Odense University Hospital, 5000 Odense, Denmark; 3 Institute for Regional Health Research, University of Southern Denmark, 5230 Odense, Denmark

**Keywords:** lung cancer, surgery, pulmonary function test, diffusing capacity, lung disease

## Abstract

**Background**: The segment count method (SCM) is standard for predicting post-operative pulmonary function tests (ppo-PFTs) in patients undergoing surgical resection for non-small cell lung cancer. Accurate prediction is pivotal for providing optimal treatment; however, earlier studies indicate that SCM underestimates ppo-PFTs. This study aimed to investigate whether this underestimation correlates with the specific lobe resected or pre-operative PFT values. **Methods**: This retrospective single-centre study evaluated patients who underwent surgical resection (wedge, segmentectomy, or lobectomy) for lung cancer. Clinical data were extracted from electronic medical files. PFTs (forced expiratory volume in the first second, FEV1, and diffusing capacity of the lungs for carbon monoxide, D_L,CO_) were performed before and within 1 year after surgery and reported as % predicted. The SCMs were calculated as the pre-operative PFTs minus the proportion related to the number of resected segments. Pre-operative D_L,CO_ was categorised as 40–60%, 61–80%, and >80%. **Results**: In total, 117 patients were included (lobectomy *n* = 102, wedge resection *n* = 15). PFTs were preserved with wedge resection. In patients undergoing lobectomy, a >10% prediction error of SCM on D_L,CO_ was observed in 74% (FEV_1_: 63%) and most often due to higher-than-predicted (D_L,CO_: 63%; FEV_1_: 52%) than lower-than-predicted PFT (D_L,CO_: 11%; FEV_1_: 11%). A higher BMI was significantly associated with lower-than-predicted PFTs. No association was found regarding the resected lobe. Notably, the prediction error of SCM increased with decreasing D_L,CO_: post-operative D_L,CO_ was preserved in patients with pre-operative D_L,CO_ 40–60%. **Conclusions**: The segment count method exhibits systematic imprecision that correlates inversely with baseline D_L,CO_ levels. Further studies are needed to validate this finding and to investigate how the SCM can be refined to reduce prediction error, especially in patients with the lowest preoperative PFTs.

## Simple Summary

Guidelines recommend using the segment count method (SCM) for prediction of post-surgical lung function (D_L,CO_ and FEV_1_) after lung cancer resection. Earlier studies indicated that a 10% prediction error of SCM concerning FEV_1_ is common. We explored the prevalence of prediction error in D_L,CO_ in a retrospective cohort. A D_L,CO_ prediction error >10% was observed in 74%, most often due to underestimation (63%, overestimation: 11%). Prediction error increased inversely with pre-surgical D_L,CO_, indicating that recovery of D_L,CO_ is larger than expected. Future studies should investigate underlying mechanisms.

## 1. Introduction

Lung cancer is the most common cause of cancer-related death, both in high- and in low-to-middle-income countries [1]. Most cases are non-small cell lung cancer (NSCLC), and surgical resection is the recommended treatment for localised NSCLC [2,3]. Video-assisted thoracoscopic surgery (VATS) resection is the preferred modality due to a lower rate of complications and post-surgical symptoms. VATS allows for anatomical (segments, lobe(s), lung) and non-anatomical (wedge) resection, and the modality depends on several aspects related to the tumour (stage, location), patient history (comorbidity, prior surgery), and patient’s preferences [2,4,5]. Impaired lung function prior to surgery is common due to shared pathophysiological pathways of lung cancer, chronic obstructive pulmonary disease (COPD), and emphysema. Correct prediction of post-operative (ppo) lung function is crucial for choice of surgical modality, as decline in lung function depends on resection extent [6,7,8].

Forced expiratory volume in the first second (FEV_1_) and diffusing capacity of the lungs for carbon monoxide (D_L,CO_) are the recommended parameters for assessing respiratory fitness prior to NSCLC surgery [9,10]. Post-operative mortality, morbidity, and complication risks significantly increase when either ppo-FEV_1_ or ppo-D_L,CO_ falls below 40%, with 40% recognised as a high-risk threshold [9,10]. Specifically, D_L,CO_ is established as a predictor op postoperative complications, days in hospital, and overall survival [11]. Therefore, the precision of ppo estimates is pivotal for accurately predicting the realistic short- and long-term risks associated with specific surgical modalities [10,12,13]. Owing to its simplicity, the Segment Count Method (SCM) remains the most frequently used approach; it requires only knowledge of pre-surgical FEV_1_ and D_L,CO_ values alongside the planned number of segments to be excised [9,10].

Measured post-surgical FEV_1_ seems to exceed ppo-FEV_1_ by approximately 10%, attributed to lung plasticity with recruitment of airways in the remaining ipsilateral lung, and resection of emphysematous lung tissue (especially with upper lobe resections) [14,15]. While longitudinal improvement of post-surgical D_L,CO_ has been reported during the first year following lobectomy [16], knowledge remains limited regarding the discrepancy between measured post-surgical D_L,CO_ and ppo-D_L,CO_, as well as the potential predictors of an unexpectedly higher /or lower) post-surgical D_L,CO_ decline.

In this study on patients undergoing planned surgical resection of NSCLC, we aimed to explore the prediction error of the SCM concerning post-surgical D_L,CO_, including assessing the prevalence of (and risk factors for) deviations >10% between measured and SCM-predicted D_L,CO_.

## 2. Materials and Methods

### 2.1. Study Design and Participants

A single-centre observational retrospective trial of patients diagnosed with localised NSCLC at our Endoscopy Unit, Zealand University Hospital Næstved, from 1 January 2018 to 31 December 2020, and referred for surgical resection at the local department of thoracic surgery. Exclusion criteria were (1) absence of postoperative pulmonary function tests, (2) performed more than one year after surgery, (3) death within three months after surgery, and (4) pneumonectomy. All PFTs in our unit are performed according to international standards [17,18].

### 2.2. Outcomes

Primary outcome: prediction error of the SCM on post-surgical D_L,CO_ within 12 months from anatomical lung cancer resection (segmentectomy, lobectomy, bilobectomy) with non-anatomical wedge resection as comparator.

Secondary outcomes: SCM prediction error on post-surgical FEV_1_, correlation of FEV_1_ and D_L,CO_ decline, impact of pre-surgical D_L,CO_ on post-surgical D_L,CO_ decline, characterisation of subjects with post-surgical D_L,CO_. lower than ppo-D_L,CO_, prevalence of SCM prediction error >10%, and risk factors for a >10% prediction error.

### 2.3. Data Collection

From electronic medical records, we extracted data on age, biological sex, height, body mass index (BMI), chronic obstructive pulmonary disease (COPD), interstitial lung disease (ILD), obesity (BMI > 40 kg/m^2^), diaphragmatic paralysis, and prior lung surgery, including the number of previously resected segments.

Presence of preoperative emphysema, postoperative full lung expansion, pleural fluid, NSCLC relapse, or other (e.g., diaphragmatic paralysis, pulmonary embolism) was assessed from pre- and post-surgical contrast-enhanced CT chest and abdomen (at the time of PFT).

The following surgical data were collected: surgery type (wedge, segmentectomy, lobectomy, bilobectomy), lobe(s) and laterality, and pTNM. Also, data on adjuvant therapy, including chemotherapy and chemoradiation, were collected.

### 2.4. Pulmonary Function Testing (PFT)

Dynamic spirometry and single-breath diffusing capacity to carbon monoxide (D_L,CO_) were performed preoperatively (during diagnostic workup 2 to 6 weeks prior to surgery) and postoperatively (within one year after surgery) according to guidelines and by trained staff using either a Jaeger MasterScreen PFT Pro system (CareFusion, Höchberg, Germany) and Jaeger MasterScreen Body (CareFusion, Höchberg, Germany) or Vyntus Body (Vyaire Medical, Hoechberg, Germany) [17,18]. The following data were extracted: FEV_1_ (L), FEV_1_ (% of predicted), FVC (% of predicted), FEV_1_/FVC ratio, D_L,CO_ (% of predicted), and haemoglobin-corrected D_L,CO_ (D_L,COc_) (% of predicted). Lung function values in percent of predicted were calculated according to standard reference equations [19]. Haemoglobin values were extracted from electronic medical records.

### 2.5. Surgical Procedures

Wedge resection, segmentectomy, lobectomy, or bilobectomy were performed according to guidelines, as video-assisted thoracic surgery (VATS) when feasible, otherwise as open thoracotomy.

### 2.6. The Segment Count Methods

Percent change in PFT before (pre) and after surgery (post) was calculated as

(1) $$\mathrm{Change}\;(\%)=\frac{\mathrm{post}-\mathrm{pre}}{\mathrm{pre}}$$

Predicted post-operative (ppo) values were calculated based on the number of excised segments (s) [10,20]:

(2) $$\mathrm{ppo}\;{\mathrm{FEV}}_{1}=\mathrm{pre}\;{\mathrm{FEV}}_{1}*(1-\frac{s}{19-\mathrm{prior}\;\mathrm{resected}\;\mathrm{segments}})$$

(3) $$\mathrm{ppo}\;D_{L,\mathrm{CO}}=\mathrm{pre}\;D_{L,\mathrm{CO}}*(1-\frac{s}{19-\mathrm{prior}\;\mathrm{resected}\;\mathrm{segments}})$$

Difference in ppo and postoperatively measured PFT was calculated as

(4) $$\mathrm{Relative}\;\mathrm{difference}\;(\%)=\frac{\mathrm{post}-\mathrm{ppo}}{\mathrm{ppo}}$$

As a quality control measure, we compared the segment-counting methods with the ppo-D_L,CO_ estimated from lung perfusion scintigraphy in the subpopulation of patients who underwent preoperative lung perfusion scintigraphy. The ppo-D_L,CO_ based on perfusion was calculated using the following formula [21,22]:

(5) $$\mathrm{ppo}\;D_{L,\mathrm{CO}}\;(\mathrm{scintigraphy})=\mathrm{pre}\;D_{L,\mathrm{CO}}*(1-(\frac{{\%\mathrm{perfusion}}_{\mathrm{right}}}{100}*\frac{s_{\mathrm{right}}}{10}+\frac{{\%\mathrm{perfusion}}_{\mathrm{left}}}{100}*\frac{s_{\mathrm{left}}}{9})),$$

where %perfusion_right_ and %perfusion_left_ represent relative perfusion, s_right_ and s_left_ denote number of resected segments, and denominators 10 and 9 correspond to total number of bronchopulmonary segments in right respectively left lung.

### 2.7. Definitions

Prediction error >10%: a ratio of measured/predicted postoperative FEV_1_ or D_L,CO_: lower (measured value <90% of predicted) or higher (>110% of predicted) than predicted.

Preoperative D_L,CO_ categories: low (40–60% of predicted), intermediate (61–80% of predicted), high (≥81% of predicted).

### 2.8. Power Calculation

The aim of the present study was exploratory, and the mean difference (or SD) between predicted and measured post-surgical values was not known prior to study onset. To detect a mean difference of 10 with SD 30 (or any other mean with three times higher SD) with 90% power (1-β) and α 0.05, we needed 97 patients with full sets of paired data.

### 2.9. Statistics

Data were analysed using R version 4.5.0 (R Foundation Statistical Computing, Vienna, Austria). Descriptive statistics were presented as mean ± standard deviation (SD) for continuous variables and as frequencies and percentages for categorical variables. Time from surgery to postoperative PFT was additionally reported as median [25th; 75th percentile]. Paired Student’s *t*-tests were used to compare within-patient pre- and postoperative outcomes and postoperative and ppo outcomes from PFT. Welch’s two-sample *t*-tests were used to compare outcomes from PFT in upper lobes with lower lobes and compare lobectomies and bilobectomies with wedge resections.

Agreement between ppo and measured postoperative values was assessed by Bland–Altman analysis. Prediction error was defined as measured minus ppo value, and systematic bias was reported as the mean difference with 95% confidence interval (CI). Precision of individual predictions was described by 95% limits of agreement (LoA, mean difference ± 1.96 SD) and mean absolute error (MAE). Proportional bias was assessed by linear regression of the difference on the mean of measured and ppo values. Pearson’s correlation coefficient (R^2^) was reported as a measure of association only. In addition, prediction error was expressed as the ratio of measured to ppo value × 100.

The association between prediction error and the preoperative value was analysed by linear regression with both variables as continuous variables. The association between prediction error and time from surgery to postoperative PFT was assessed by linear regression and Spearman’s rank correlation, and a sensitivity analysis was restricted to patients with postoperative PFT performed 90–180 days after surgery.

Comparisons between performance groups and groups based on preoperative D_L,CO_ values were conducted using one-way ANOVA for continuous variables with Tukey’s honest significant differences test for post hoc pairwise comparisons, when appropriate. The chi-squared test was used to analyse differences in categorical variables. Statistical significance was set at *p* < 0.05 for all analyses.

## 3. Results

### 3.1. Study Population

**Figure 1:**
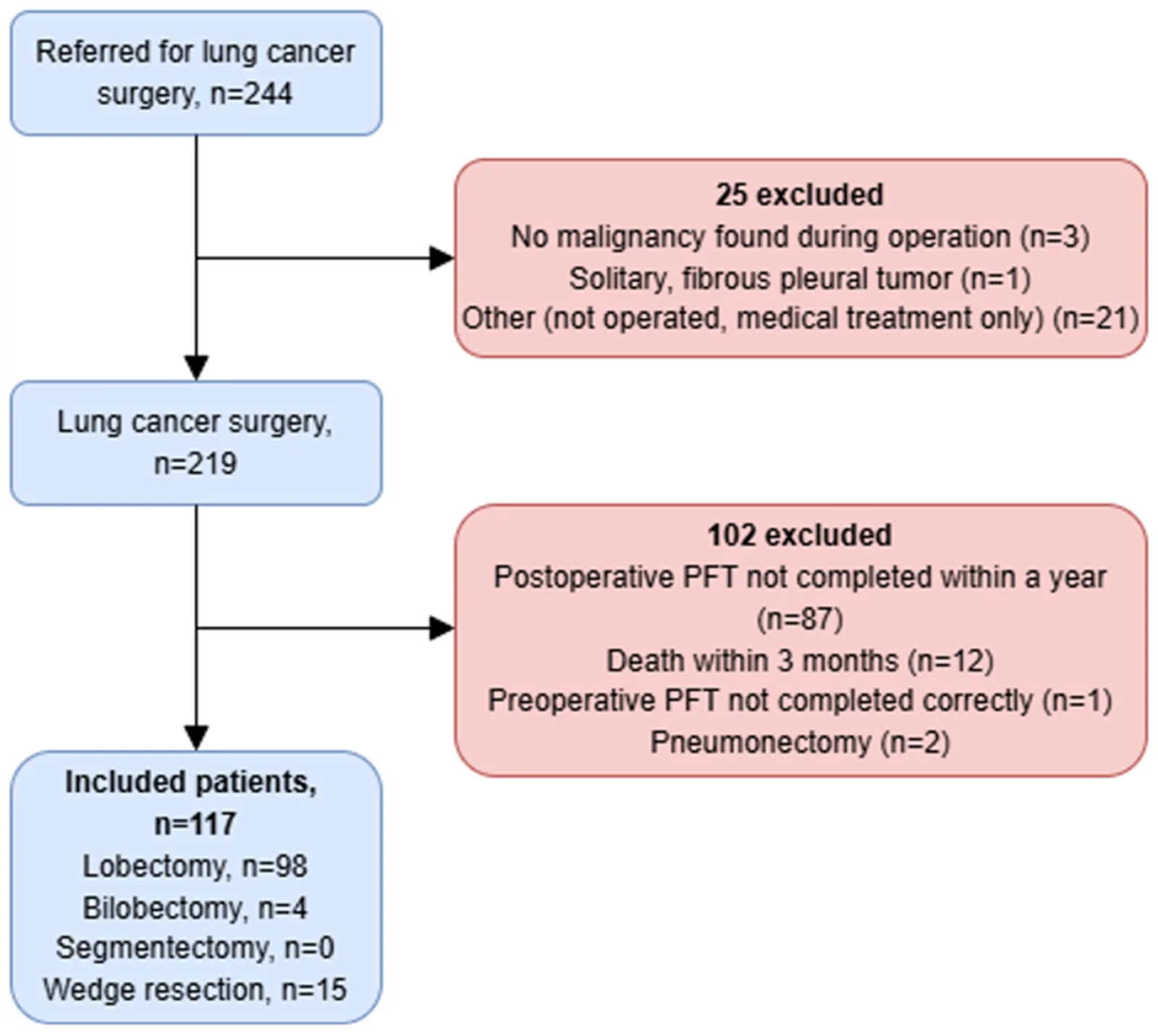
Flow chart of included participants.

We included a total of 117 patients (see Figure 1), of whom 82 (70%) were diagnosed with stage I lung cancer, 17 (15%) with stage II, and 18 (15%) with stage III NSCLC (see Supplementary Table S1 for further details on TNM and histopathology). Baseline characteristics of the study population are presented in Table 1. Post-surgical CT control at the time of PFT showed full lung expansion in the vast majority, with only minor post-surgical pleural effusion; see Supplementary Table S2.

### 3.2. Lung Function Before and After Surgery

PFT was performed 32 ± 24 days before surgery and 136 ± 64 days after surgery. Post-surgical decline in PFT was similar across lobes resected, and PFT was unchanged after wedge resection; see Supplementary Figure S1 and Supplementary Table S3. We observed no difference between D_L,CO_ and haemoglobin-corrected D_L,CO_ (D_L,Coc_), see Supplementary Table S3. Time from resection to PFT did not impact the prediction error of FEV_1_ results, but for D_L,CO_ the prediction error increased. A sensitivity analysis including the 64 patients (63%) who underwent PFT between 3 and 6 months (i.e., plateau of post-surgical FEV_1_ recovery) after resection showed a strikingly similar pattern. The measured/ppo-D_L,CO_ ratio was 112% (SD 15) in the sensitivity cohort versus 114% (SD 19) in the overall cohort (*n* = 102), and 111% (SD 18) versus 113% (SD 19) for FEV_1_.

Concerning upper versus lower lobectomy, we found no significant differences in either absolute PFT values or percentage change (Welch’s *t*-test all > 0.05).

Post-surgical decline in FEV_1_ largely followed that of the decline in D_L,CO_, except for a larger FEV_1_ than D_L,CO_ loss with upper lobe resection (*p* = 0.019); see Supplementary Figure S2.

Perfusion scintigraphy was performed in 23 cases. Correction of the segment count method for scintigraphic distribution had negligible impact on ppo-D_L,CO_ % (see Supplementary Figure S3 and Supplementary Table S4).

### 3.3. Segment Count Method Prediction Error

**Figure 2:**
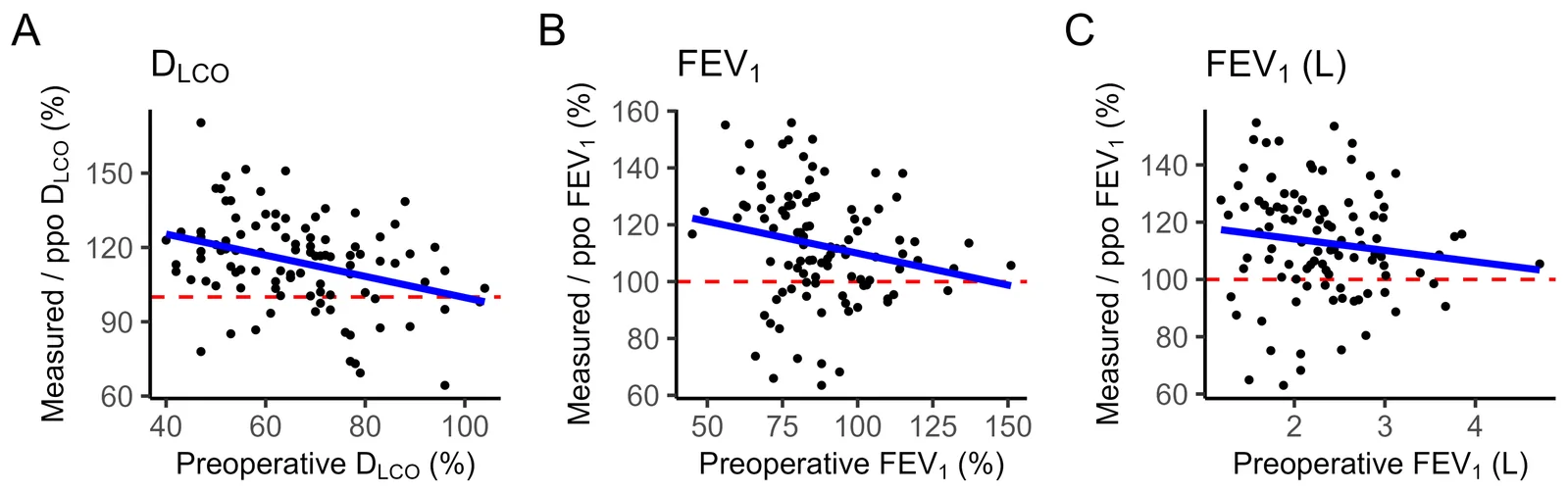
Prediction error, expressed as measured/predicted postoperative value × 100, plotted against the preoperative value ((**A**) D_L,CO_ (diffusing capacity for carbon monoxide) % predicted; (**B**) FEV_1_ (forced expiratory volume in the first second) % predicted; (**C**) FEV_1_ in litres) for lobectomies or bilobectomies (*n* = 102). The blue line indicates the linear regression, the red dotted line indicates perfect prediction (100%), and black dots indicate each participant. Values above 100% indicate higher-than-predicted measured postoperative function.

Measured postoperative PFTs were consistently higher than the predicted values (see Table 2). Figure 2 shows that prediction error was inversely associated with preoperative PFTs; thus, prediction error was largest with the lowest preoperative D_L,CO_ or FEV_1_.

**Figure 3:**
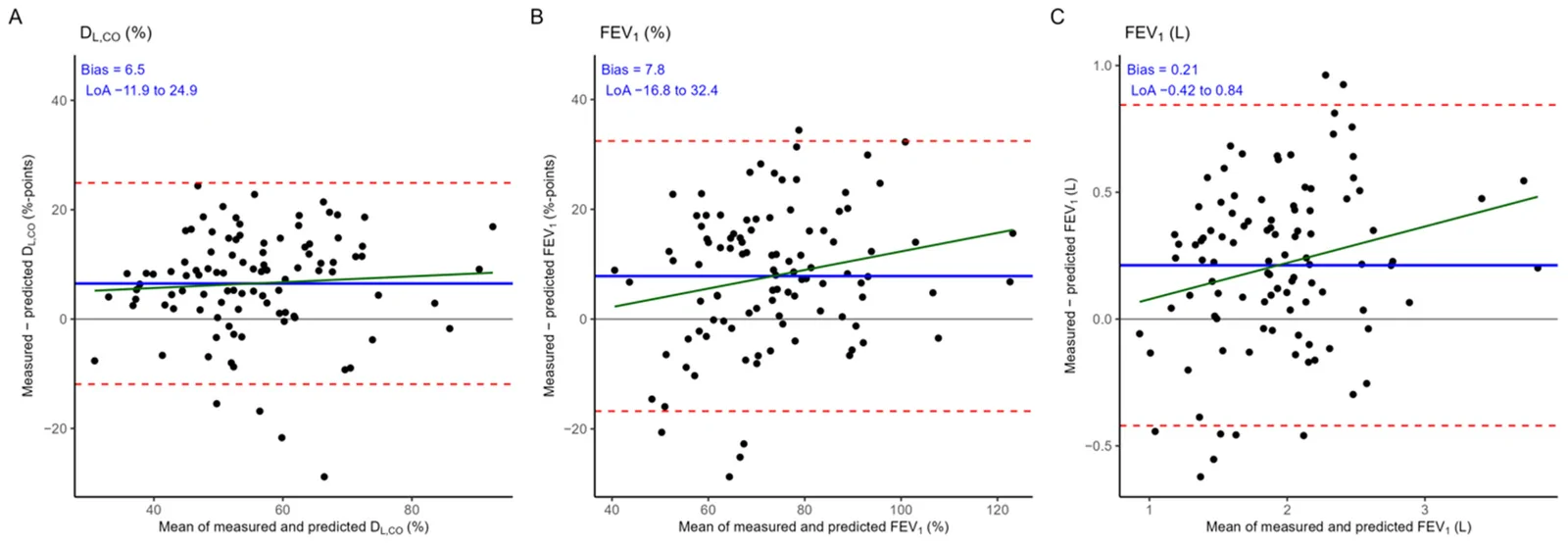
Bland–Altman plots of prediction error (measured minus ppo value) versus the mean of measured and ppo values in lobectomies and bilobectomies (*n* = 102). (**A**) D_L,CO_ (diffusing capacity for carbon monoxide) % predicted, (**B**) FEV1% predicted, and (**C**) FEV1 (forced expiratory volume in the first second) in litres. Black dots indicate each participant. The solid blue line indicates the mean bias (mean difference between measured and ppo value), and the red dashed lines indicate the 95% limits of agreement (LoA; mean bias ± 1.96 SD), within which approximately 95% of individual prediction errors are expected to fall. Values of bias and LoA are given in blue in each panel. The grey line indicates zero difference (perfect prediction), and the green line indicates the linear regression of the difference on the mean (proportional bias). Positive values indicate higher-than-predicted measured postoperative function.

The mean absolute error of SCM for post-surgical D_L,CO_ was 9.6 percentage points (FEV_1_% predicted: 12.1% percentage points), and Bland–Altman plots (Figure 3) show a constant systematic, positive bias of SCM for D_L,CO_ regardless of post-operative D_L,CO_ level. For FEV_1,_ bias is proportional to increasing post-operative FEV_1_ volume or % predicted.

In the majority of patients, the SCM prediction error exceeded >10% (D_L,CO_: 74%; FEV_1_: 63%) and was mostly due to >10% higher-than-predicted measured post-surgical PFT (D_L,CO_: 63%; FEV_1_: 52%) than lower-than-predicted PFT (D_L,CO_: 11%; FEV_1_: 11%), see Supplementary Table S5A,B. This table also shows that lower-than-predicted FEV_1_ and D_L,CO_ were mutually associated, and that BMI (but not gender, age, or underlying lung disease) was significantly associated with lower-than-predicted FEV_1_ and D_L,CO_.

### 3.4. Lung Function Changes Stratified by Preoperative D_L,CO_

Across all pre-operative D_L,CO_ categories, measured post-operative D_L,CO_ was higher than ppo values (yet only statistically significant in patients with pre-operative D_L,CO_ ≤ 80%). The SCM prediction error increased with decreasing pre-operative D_L,CO_, resulting in an insignificant post-surgical decrease D_L,CO_ in patients with pre-operative D_L,CO_ ≤ 60% (see Table 3).

## 4. Discussion

The present paper suggests that the accuracy of the widely used segment count method for prediction of post-operative PFT, and in particular D_L,CO_, after VATS lobectomy for localised NSCLC depends on the preoperative D_L,CO_. Reduced PFT is commonly found in patients diagnosed with NSCLC due to shared pathophysiological pathways (tobacco smoke exposure and epigenetic risk factors) in the development of emphysema, COPD, and lung cancer [23]. In the setting of surgically resectable lung cancer, correct prediction of post-operative lung function is pivotal, as surgery is considered safe if ppo values are above 40%, whereas ppo values below 30% are associated with severely impaired quality of life [9].

Lobectomy is still the most common resection procedure for localised lung cancer when the more lung-sparing procedures such as segmentectomy or wedge resection are not feasible or available [7,24]. Both the most recent American (American College of Chest Physicians) and European (European Respiratory Society and European Society of Thoracic Surgery) clinical guidelines recommend the segment count method as standard in case of lobectomy to predict post-operative FEV_1_ and D_L,CO_ [10,20]. Other methods to predict post-operative FEV_1_ or D_L,CO_ do exist, such as ventilation and/or perfusion scintigraphy, or quantitative CT, magnetic resonance imaging (MRI), single photon emission computed tomography (SPECT), or in combination [9,10]. Yet the recommendation is that ventilation or perfusion scintigraphy is used only before pneumonectomy (due to inaccuracy in translating scintigraphy values to resected segments at lobectomy), quantitative CT scan is used only in patients with borderline lung function, and the remaining techniques are only for research [9,10].

In the present study, we measured PFT approximately 4 months after surgical resection; however, post-operative lung volume and lung function change with time since lobectomy, which is at its lowest in the first weeks after surgery. Only a few patients underwent Wedge resection, with no significant loss in PFTs, as observed in larger studies [25]. Two recent large Chinese and Japanese studies both showed steady improvement in post-lobectomy FEV_1_ in the first 12 months after surgery [14,26]. The latter study also explored the role of BMI and found a positive association between increasing BMI and increasing mismatch between predicted and measured ppo-FEV_1_ [14]. A smaller Korean study from 2016 found that post-lobectomy FEV_1_ continued to increase during 12 months, reaching the ppo-FEV_1_ values assessed by lung perfusion [16]. The same study additionally investigated the recovery of D_L,CO_ which continued to increase during the 12 months and surpassed the ppo-D_L,CO_ by >20% [16]. However, the external validity of these findings can be questioned as the Korean population was both younger than ours (63 versus 69 years) and had markedly higher pre-operative PFT (FEV_1_% predicted 99% vs. 88%; D_L,CO_ % predicted 84% versus 66%), perhaps reflecting that non-smoking-related, EGFR-mutated lung cancers are more common in South-East Asia than in Europe [27].

Other studies using the segment count method found that measured post-operative FEV_1_ exceeded ppo-FEV_1_ by around 10%, but with clear differences depending on lobe: RML 3%, RUL 4%, LUL 10%, LLL 21% and RLL 26% [14,28]. A pattern of lobe-dependent heterogeneity in lung recovery was elegantly demonstrated in a recent Chinese study using serial lung volume assessment at volumetric chest CT, showing that all non-resected lobes expand post-surgically but with ipsilateral predominance, and with the largest reserve in the lower lobes [26]. We did not find any clear patterns concerning resected lobe and SCM prediction error, but observed that time from resection was positively associated with higher D_L,CO_ prediction error. This may be due to lack of “steady state,” as PFT continues to increase up to 12 months post-surgically. Thus, we cannot explain the higher-than-predicted post-surgical FEV_1_ or D_L,CO_ with resection of hyperinflated, emphysematous upper lobes, but a similar mechanism of lobectomy in patients with lower D_L,CO_ may be present: recruitment of alveolar reserve in remaining lobes, and perhaps even improvement in diaphragmatic excursions [29,30].

Our study has both strengths and limitations. In our unit, we routinely measure post-surgical PFT as part of follow-up after surgery [31]. The non-experimental, retrospective design and use of everyday clinical data had the disadvantage of several patients missing post-surgical PFT with too late or absent reminders. We decided to not use data imputation to avoid introducing serious bias, as we did not know whether non-performed post-surgical PFTs were a random or systematic phenomenon [32]. As a routine in our unit, all lung function tests are performed according to international guidelines [17,18], and whenever possible, serial PFTs are performed on the same equipment. However, medical records lacked information if same equipment was used for post-operative PFT or not. The retrospective nature also prohibited collection of non-routine data such as structured patient-reported outcomes on symptoms or quality of life, which are relevant to investigate whether the observed differences in post-operative D_L,CO_ decline were clinically relevant [33]. We excluded patients without post-surgical PFT, and this selection may introduce a survivor bias by including only those who were fit enough to attend and complete the PFT. This could potentially bias patient inclusion towards an over-representation of better-than-expected PFTs, which in turn would skew our conclusion towards a higher positive prediction error. Similarly, we did not include data on surgical procedure, tumour size, or possible adjuvant therapy, which could potentially impact the measured PFT. However, the data set was too small for extensive regression models on associations with prediction error, and our focus was mainly to explore the existence of a possible prediction error. Also, the segment count method inherently implies mathematical coupling since ppo-PFTs are calculated directly from the pre-surgical values. By focusing on relative, not absolute, post-surgical changes, and the observed difference in prediction error of D_L,CO_ and FEV_1_ suggests a limited impact of mathematical coupling as an explanation for the observed results [34].

Another limitation is that we performed multiple subgroup comparisons without adjusting for multiple testing. However, this is not formally required with exploratory studies, and the signal on prediction error of ppo-D_L,CO_ was robust across different analyses. Furthermore, we did not subtract the number of obstructed segments from the SCM equation. Including obstructed segments invariably implies an exaggeration of the decrease in ppo-PFT values. However, we find it unlikely that this explains the observed findings, since the majority underwent surgery for stage I lung cancer, where segment obstruction is rare, and correction for perfusion scintigraphic distribution had almost no impact on ppo-D_L,CO_ values [10,20]. Scintigraphic data were only available in a subgroup of patients; however, these were most likely selected cases where a scintigraphy was needed to ensure fitness for surgery. Lastly, we did not include patients with ppo-PFT <40%, which is the cutoff for high risk for surgical resection in the latest guidelines. Extrapolation of our results to patients with ppo-PFT < 40% should only be considered with extreme caution [10].

We suggest that a future prospective study clarify the prediction error concerning D_L,CO_ 12 months after resection of different prediction methods (including the segment count method) and regardless of pre-surgical D_L,CO_, ppo-PFTs, or surgical method (including robot-assisted surgery), e.g., by combining with quantitative CT, so future assessment of fitness for lung cancer resection is based on the most precise prediction [8,10]. A prospective study will also allow for exploration of mechanisms behind the slow but steady improvement in the first year after resection, and allow for multivariable analyses of risk factors for both worse- and better-than-expected post-surgical PFTs [29,30].

## 5. Conclusions

In conclusion, our data supports earlier findings that the segment count method underestimates post-operative PFT. Our data indicate that this prediction error is more pronounced with D_L,CO_ than FEV_1_, and especially in patients with D_L,CO_ ≤ 80%, while increasing BMI was associated with lower-than-expected PFTs. Future studies should explore how to reduce prediction errors, as the segment count method is otherwise attractive due to its simplicity.

## Figures and Tables

**Table 1:** Baseline characteristics.

	Lobectomy (*n* = 98) or Bilobectomy (*n* = 4)	Wedge Resection
Number of participants	102	15
Female (*n*, %)	61 (60%)	8 (53%)
Age (years)	69 ± 9	73 ± 7
Height (cm)	170 ± 9	168 ± 10
BMI (kg/m^2^)	27 ± 5	26 ± 6
**Surgery type (*n*, %)**		
VATS	88 (86%)	13 (87%)
Thoracotomy	14 (14%)	2 (13%)
**Prior surgery (*n*, %)**		
Wedge	0 (0%)	1 (7%)
Lobectomy	0 (0%)	2 (13%)
**Lobe resected (*n*, %)**		
Right upper lobe (RUL)	33 (32%)	
Right middle lobe (RML)	9 (9%)	4 (27%)
Right lower lobe (RLL)	19 (19%)	0 (0%)
Left upper lobe	18 (18%)	4 (27%)
Left lower lobe	19 (19%)	3 (20%)
RUL + RML	2 (2%)	4 (27%)
RML + RLL	2 (2%)	
**Adjuvant therapy (*n*, %)**		
Systemic treatment	20 (20%)	2 (13%)
Radiation	4 (4%)	0 (0%)
**Comorbidities (*n*, %)**		
Chronic obstructive lung disease	21 (20%)	3 (20%)
Interstitial lung disease	1 (1%)	0 (0%)
Body mass index >40	3 (3%)	0 (0%)
Diaphragmatic paralysis	0 (0%)	0 (0%)
Other	4 (4%)	2 (13%)
**CT scan (*n*, %)**		
Emphysema	55 (54%)	9 (60%)
If yes: D_L,CO_ < 70% pred	46 (45%)	8 (53%)

Abbreviations: VATS: video-assisted thoracic surgery; CT: computed tomography; D_L,CO_: diffusing capacity for carbon monoxide.

**Table 2:** Measured vs. predicted postsurgical FEV_1_ or D_L,CO_ (% predicted).

	After Surgery	ppo	*p* Value	Percentage Difference
All lobectomies and bilobectomies (*n* = 102)				
FEV_1_ (L)	2.04 ± 0.59	1.83 ± 0.51	**	13 (9; 17)
FEV_1_ (% predicted)	77 ± 18	70 ± 15	**	13 (9; 17)
D_L,CO_ (% predicted)	59 ± 13	52 ± 13	**	14 (11; 18)
**Upper lobe resection (*n* = 53)**				
FEV_1_ (L)	2.03 ± 0.53	1.88 ± 0.45	*	9 (4; 14)
FEV_1_ (% predicted)	77 ± 19	71 ± 15	**	9 (4; 14)
D_L,CO_ (% predicted)	57 ± 11	51 ± 11	**	14 (9; 18)
**Lower lobe resection (*n* = 49)**				
FEV_1_ (L)	2.05 ± 0.65	1.77 ± 0.58	**	17 (12; 23)
FEV_1_ (% predicted)	77 ± 17	67 ± 16	**	17 (11; 23)
D_L,CO_ (% predicted)	61 ± 15	54 ± 14	**	15 (9; 21)
**Wedge resection (*n* = 15)**				
FEV_1_ (L)	1.86 ± 0.65	1.89 ± 0.47		0 (−7; 7)
FEV_1_ (% predicted)	77 ± 17	78 ± 18		1 (−6; 8)
D_L,CO_ (% predicted)	64 ± 15	64 ± 13		2 (−8; 13)

Abbreviations: ppo: predicted postoperative; FEV_1_: forced expiratory volume in the first second; D_L,CO_: diffusing capacity for carbon monoxide. *t*-test measured versus ppo values: * *p*-value < 0.01, ** <0.001.

**Table 3:** Post-surgical decline in D_L,CO_ stratified by pre-surgical D_L,CO_ in patients undergoing lobectomy (*e* = 102).

	Group 1 D_L,CO_ 40–60%	Group 2 D_L,CO_ 61–80%	Group 3 D_L,CO_ >80%	*p*-Value for Trend ^§^
Number of participants	40	46	16	
Resected segments	4 ± 1	4 ± 1	4 ± 1	0.932
**FEV_1_ (% predicted)**				
Pre-FEV_1_ (% pred)	86 ± 18	89 ± 16	90 ± 27	0.592
Post FEV_1_	76 ± 18 **	78 ± 18 **	80 ± 20 *	0.799
ppo-FEV_1_	68 ± 15 ^##^	70 ± 14 ^##^	71 ± 21 ^#^	0.724
Change in FEV_1_ (%)				
** **(Post-pre)/pre	−10 (−15; −6)	−12 (−16; −8)	−10 (−18; −1)	0.724
Relative difference in FEV_1_ (%)				
** **(Post-ppo)/ppo	13 (7; 20)	11 (6; 17)	15 (4; 26)	0.783
**D_L,CO_ (% predicted)**				
Pre-D_L,CO_ (% pred)	51 ± 5	70 ± 6	91 ± 7	**<0.001**
Post-D_L,CO_	49 ± 9	61 ± 9 **	77 ± 12 **	**<0.001**
ppo-D_L,CO_	41 ± 5 ^##^	55 ± 6 ^##^	73 ± 10	**<0.001**
Change in D_L,CO_ (%)				
** **(Post-pre)/pre	−4 (−8; 0.4)	−13 (−17; −9)	−16 (−23; −10)	**0.002**
Relative difference in D_L,CO_ (%)				
** **(Post-ppo)/ppo	21 (16; 27)	11 (6; 16)	7 (−3; 17)	**0.006**

Abbreviations: ppo: predicted postoperative; FEV_1_: forced expiratory volume in the first second; D_L,CO_: diffusing capacity for carbon monoxide. *t*-test ppo versus post values: * *p*-value < 0.05, ** <0.001, ^#^ <0.05 and ^##^ <0.001. ^§^ one-way ANOVA.

## Data Availability

All data generated and analysed during this study are included in this article and its Supplementary Material files. Further enquiries can be directed to the corresponding author.

## Supplementary Materials

The following supporting information can be downloaded at https://www.mdpi.com/article/10.3390/cancers18213576/s1: Figure S1: Pre- and post-operative FEV_1_ and D_L,CO_ after lobectomy and wedge resection; Figure S2: Scatterplot on absolute pre- to post-surgery changes in FEV_1_ compared to D_L,CO_; Figure S3: ppo-D_L,CO_ (segment count) vs. ppo-D_L,CO_ (perfusion); Table S1: Cancer characteristics; Table S2: Results of post-surgical contrast-enhanced control CT at the time of PFT; Table S3: Pulmonary function test values before and after surgery; Table S4: V/Q scintigraphy; Table S5A,B: Characteristics of patients stratified according to ratio of measured versus predicted post-operative FEV_1_ (A), and D_L,CO_ (B).
