# Supplementary material for: Measured Postoperative DL,CO and FEV1 Frequently Exceed Segment-Count Predictions After Lung Cancer Surgery: A Retrospective Cohort Study
Source: Cancers (Basel). 2026 Nov 6;18(21):3576. doi: 10.3390/cancers18213576 (PMC13761532; doi:10.3390/cancers18213576)
Supplement: Supplementary file 1 [file cancers-18-03576-s001.zip › cancers-4573409-supplementary.pdf]

Supplementary Materials

Measured Postoperative $DL_{,CO}$ and FEV_1 Frequently Exceed Segment-Count Predictions After Lung Cancer Surgery: A Retrospective Cohort Study

Table S1. Cancer characteristics.

	Lobectomies or bilobectomies (*n*=102)	Wedge resection (*n*=15)
**Diagnoses (*n*, %)**		
Adenocarcinoma	70 (69 %)	6 (40 %)
Squamous cell carcinomas	22 (22 %)	5 (33 %)
Other	10 (10 %)	4 (27 %)
Two synchronous lung cancers	3 (3 %)	2 (13 %)
**pTNM stage (*n*, %)**		
IA1	4 (4 %)	2 (13 %)
IA2	25 (25 %)	3 (20 %)
IA3	17 (17 %)	2 (13 %)
IB	27 (26 %)	2 (13 %)
IIA	1 (1 %)	0 (0 %)
IIB	13 (13 %)	3 (20 %)
IIIA	13 (13 %)	3 (20 %)
IIIB	2 (2 %)	0 (0 %)

Categorical outcomes reported as number (percentage).

Table S2. Results of post-surgical contrast-enhanced control CT at the time of PFT.

	**Lobectomies or bilobectomies (*n*=102)**	**Wedge resection (*n*=15)**
Lung not fully expanded (*n*, %)	5 (5 %)	2 (13 %)
Degree of non-expansion:		
Small (<1/3 of hemithorax)	4 (4 %)	2 (13 %)
Moderate (1/3 to 2/3 of hemithorax)	1 (1 %)	0 (0 %)
Large (>2/3 of hemithorax)	0 (0 %)	0 (0 %)
Pleural effusion (*n*, %)	42 (41 %)	6 (40 %)
Effusion size:		
Small (<1/3 of hemithorax)	37 (36 %)	6 (40 %)
Moderate (1/3 to 2/3 of hemithorax)	4 (4 %)	0 (0 %)
Large (>2/3 of hemithorax)	1 (1 %)	0 (0 %)
Recurrence (*n*, %)	7 (7 %)	2 (13 %)

Categorical outcomes reported as number (percentage). Abbreviations: n: number.

Table S3. Pulmonary function test values before and after surgery.

	Before surgery	After surgery	P value	Percentage change
**All lobectomies or bilobectomies (*n*=102)**				
FEV_1 (L)	2.31 ± 0.59	2.04 ± 0.59	**	-11 (-14; -8)
FEV_1 (% pred)	88 ± 18	77 ± 18	**	-11 (-14; -8)
FVC (% pred)	95 ± 19	86 ± 19	**	-9 (-12; -6)
FEV_1/FVC ratio	0.72 ± 0.08	0.70 ± 0.08	**	-3 (-4; -1)
$D_{L,CO}$ (% pred)	66 ± 15	59 ± 13	**	-10 (-13; -7)
$D_{L,COc}$ (% pred)	66 ± 15	59 ± 13	**	-10 (-13; -7)
**Upper lobe resection (*n*=53)**				
FEV_1 (L)	2.35 ± 0.54	2.03 ± 0.53	**	-13 (-17; -10)
FEV_1 (% pred)	89 ± 18	77 ± 19	**	-13 (-17; -10)
FVC (% pred)	95 ± 18	87 ± 21	**	-10 (-14; -5)
FEV_1/FVC ratio	0.73 ± 0.08	0.70 ± 0.08	**	-4 (-6; -2)
$D_{L,CO}$ (% pred)	63 ± 14	57 ± 11	**	-9 (-13; -5)
$D_{L,COc}$ (% pred)	64 ± 14	57 ± 11	**	-9 (-13; -5)
**Lower lobe resection (*n*=49)**				
FEV_1 (L)	2.27 ± 0.73	2.05 ± 0.65	**	-9 (-13; -4)
FEV_1 (% pred)	86 ± 19	77 ± 17	**	-9 (-14; -5)
FVC (% pred)	96 ± 19	86 ± 17	**	-8 (-13; -4)
FEV_1/FVC ratio	0.71 ± 0.08	0.70 ± 0.09		-2 (-4; 1)
$D_{L,CO}$ (% pred)	68 ± 16	61 ± 15	**	-11 (-15; -6)
$D_{L,COc}$ (% pred)	69 ± 16	61 ± 15	**	-10 (-15; -6)
**Wedge resection (*n*=15)**				
FEV_1 (L)	1.89 ± 0.73	1.86 ± 0.65		0 (-7; 7)
FEV_1 (% pred)	78 ± 19	77 ± 17		1 (-6; 8)
FVC (% pred)	90 ± 19	89 ± 17		0 (-11; 12)
FEV_1/FVC ratio	0.67 ± 0.08	0.67 ± 0.09		2 (-6; 9)
$D_{L,CO}$ (% pred)	64 ± 16	64 ± 15		2 (-8; 13)
$D_{L,COc}$ (% pred)	64 ± 16	64 ± 15		3 (-8; 13)

Outcomes reported as mean ± standard deviation and percentage change as mean percentage difference (lower confidence interval; upper confidence interval). ** Student's t-test <0.001 (pre- versus post-values). Abbreviations: FEV_1: forced expiratory volume in the 1st second; FVC: forced vital capacity; $D_{L,CO}$: diffusing capacity for carbon monoxide; $D_{L,COc}$: haemoglobin-corrected diffusing capacity for carbon monoxide.

A subset of "After surgery" variables are also shown in Table 2.

Table S4. V/Q scintigraphy.

	**Lobectomies or bilobectomies (n=23)**
**Lobe resected (*n*, %)**	
RUL	7 (30 %)
RML	2 (9 %)
RLL	5 (22 %)
LUL	2 (9 %)
LLL	5 (22 %)
RUL + RML	1 (4 %)
RML + RLL	1 (4 %)
**Perfusion (percentage)**	
V/Q right upper	22 ± 6
V/Q right lower	31 ± 5
V/Q left upper	22 ± 4
V/Q left lower	26 ± 9
$D_{L,CO}$ pre (% pred)	54 ± 13
ppo-$D_{L,CO}$ (segment-count) (% pred)	42 ± 12
ppo-$D_{L,CO}$ (perfusion) (% pred)	43 ± 13

Categorical outcomes reported as number (percentage), continuously outcomes as mean ± standard deviation. Abbreviations: ; RUL: right upper lobe; RML: right middle lobe; RLL: right lower lobe; LUL: left upper lobe; LLL: left lower lobe; V/Q: ventilation-perfusion; $D_{L,CO}$: diffusing capacity for carbon monoxide.

Table S5. A: Characteristics of patients who underwent lobectomy/bilobectomy (*n*=102) and stratified according to ratio of measured versus predicted post-operative FEV_1.

**Measured post-FEV1:**	**lower (<90%) than predicted**	**as (90-110%) predicted**	**higher (>110%) than predicted**	**p-value**
Number of participants	11 (11 %)	38 (37 %)	53 (52 %)	
Female (n, %)	7 (64 %)	23 (61 %)	31 (58 %)	0.945
Age (years)	69 ± 6	68 ± 10	69 ± 9	0.846
Height (cm)	171 ± 9	169 ± 9	171 ± 9	0.645
BMI (kg/m^2)	32 ± 5	25 ± 5	27 ± 5	**0.001**
**Surgery type (*n*, %)**				
VATS	9 (82 %)	34 (89 %)	45 (85 %)	0.742
Thoracotomy	2 (18 %)	4 (11 %)	8 (15 %)	
**Lobe resected (*n*, %)**				
Upper lobe	7 (64 %)	23 (61 %)	23 (43 %)	0.195
Lower or middle lobe	4 (36 %)	15 (39 %)	30 (57 %)	
**Comorbidities (*n*, %)**				
COPD	3 (27 %)	7 (18 %)	11 (21 %)	0.814
ILD	0 (0 %)	1 (3 %)	0 (0 %)	0.427
BMI >40	1 (9 %)	0 (0 %)	2 (4 %)	0.254
Other	0 (0 %)	2 (5 %)	2 (4 %)	0.729
Emphysema on CT (*n*, %)	5 (45 %)	20 (53 %)	30 (57 %)	0.780
+ $D_{L,CO}$ <70 % pred (*n*, %)	4 (36 %)	16 (42 %)	26 (49 %)	0.666
**Pulmonary function test**				
Ratio (post/ppo)	77.4 ± 9.9	102.0 ± 5.7	128.0 ± 11.8	*na*
Pre-FEV_1 (% pred)	81 ± 11	96 ± 18	83 ± 18	**<0.001**
Post-FEV_1 (% pred)	51 ± 9	79 ± 16	81 ± 17	**<0.001**
Pre-$D_{L,CO}$ (% pred)	64 ± 17	67 ± 14	65 ± 16	0.790
Post-$D_{L,CO}$ (% pred)	45 ± 5	60 ± 12	60 ± 13	**0.001**

Categorical outcomes reported as number (percentage), continuous outcomes as mean ± standard deviation. One-way ANOVA was used to calculate p-values for continuous variables and chi squared test for categorical variables. Abbreviations: BMI: body mass index; VATS: video-assisted thoracic surgery; RUL: right upper lobe; RML: right middle lobe; RLL: right lower lobe; LUL: left upper lobe; LLL: left lower lobe; COPD chronic obstructive pulmonary disease; ILD: interstitial lung disease; CT: computed tomography; $D_{L,CO}$: diffusing capacity for carbon monoxide; FEV_1: Forced expiratory volume in the 1st second.

Table S5. B: Characteristics of patients who underwent lobectomy/bilobectomy (n=102) and stratified according to ratio of measured versus predicted post-operative $D_{L,CO}$.

**Measured post-$D_{L,CO}$:**	**lower (<90%) than predicted**	**as (90-110%) predicted**	**higher (>110%) than predicted**	**p-value**
Number of participants	11 (11 %)	27 (26 %)	64 (63 %)	
Female (n, %)	6 (55 %)	20 (74 %)	35 (55 %)	0.211
Age (years)	70 ± 6	70 ± 8	68 ± 10	0.542
Height (cm)	172 ± 11	169 ± 10	171 ± 9	0.535
BMI (kg/m^2)	31 ± 6	27 ± 5	26 ± 5	**0.011**
**Surgery type (*n*, %)**				
VATS	10 (91 %)	22 (81 %)	56 (88 %)	0.669
Thoracotomy	1 (9 %)	5 (19 %)	8 (13 %)	
**Lobe resected (*n*, %)**				
Upper lobe	5 (45 %)	15 (56 %)	33 (52 %)	0.848
Lower lobe	6 (55 %)	12 (44 %)	31 (48 %)	
**Comorbidities (*n*, %)**				
COPD	2 (18 %)	6 (22 %)	13 (20 %)	0.958
ILD	0 (0 %)	0 (0 %)	1 (2 %)	0.741
BMI >40	1 (9 %)	0 (0 %)	2 (3 %)	0.319
Other	0 (0 %)	0 (0 %)	4 (6 %	0.291
Emphysema on CT (n, %)	4 (36 %)	16 (59 %)	35 (55 %)	0.430
+ $D_{L,CO}$ <70 % pred (n, %)	2 (18 %)	12 (44 %)	32 (50 %)	0.146
**Pulmonary function test**				
Ratio (post/ppo)	79.7 ± 8.3	103.0 ± 5.0	125.0 ± 11.9	*na*
Pre-FEV_1 (% pred)	85 ± 17	92 ± 22	86 ± 17	0.328
Post-FEV_1 (% pred)	59 ± 13	78 ± 22	80 ± 15	**0.001**
Pre-$D_{L,CO}$ (% pred)	74 ± 15	70 ± 15	63 ± 14	**0.015**
Post-$D_{L,CO}$ (% pred)	48 ± 11	58 ± 11	61 ± 13	**0.009**

Categorical outcomes reported as number (percentage), continuous outcomes as mean ± standard deviation. One-way ANOVA was used to calculate p-values for continuous variables and chi squared test for categorical variables. Abbreviations: BMI: body mass index; VATS: video-assisted thoracic surgery; RUL: right upper lobe; RML: right middle lobe; RLL: right lower lobe; LUL: left upper lobe; LLL: left lower lobe; COPD chronic obstructive pulmonary disease; ILD: interstitial lung disease; CT: computed tomography; $D_{L,CO}$: diffusing capacity for carbon monoxide; FEV_1: Forced expiratory volume in the 1st second.

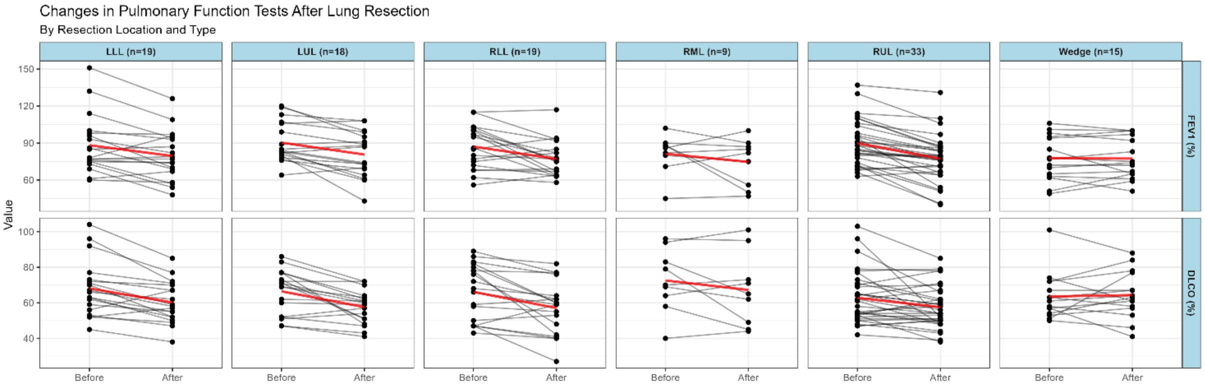

Figure S1. pre- and post-operative FEV_1 and DL_{Co} after lobectomy and wedge resection.

Lung function before and after surgery divided in lobes and wedge resection as control. Black dot with line indicates each participant and red line the mean value. Abbreviations: RUL: right upper lobe; RML: right middle lobe; RLL: right lower lobe; LUL: left upper lobe; LLL: left lower lobe; FEV_1: forced expiratory volume in the 1st second; $D_{L,CO}$: diffusing capacity for carbon monoxide.

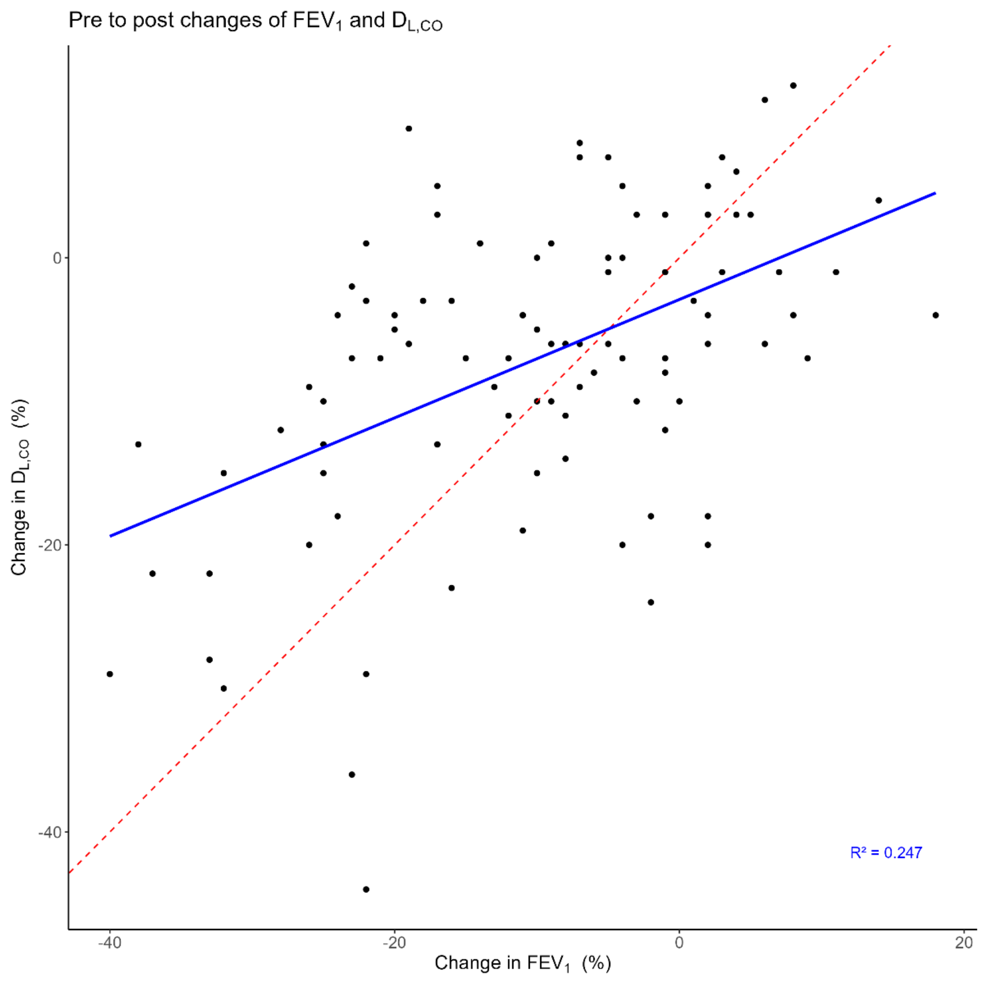

Figure S2. Scatterplot on absolute pre- to post-surgery changes in FEV1 compared to DLCO.

Linear regression of the absolute change in the actual and postoperative FEV_1 and $D_{L,CO}$ for lobectomies and bilobectomies (*n*=102). Red line: expected 1:1 change in FEV_1 and $D_{L,CO}$. Black dots represent each participant. Abbreviations: FEV_1: forced expiratory volume in the 1st second; $D_{L,CO}$: diffusing capacity for carbon monoxide.

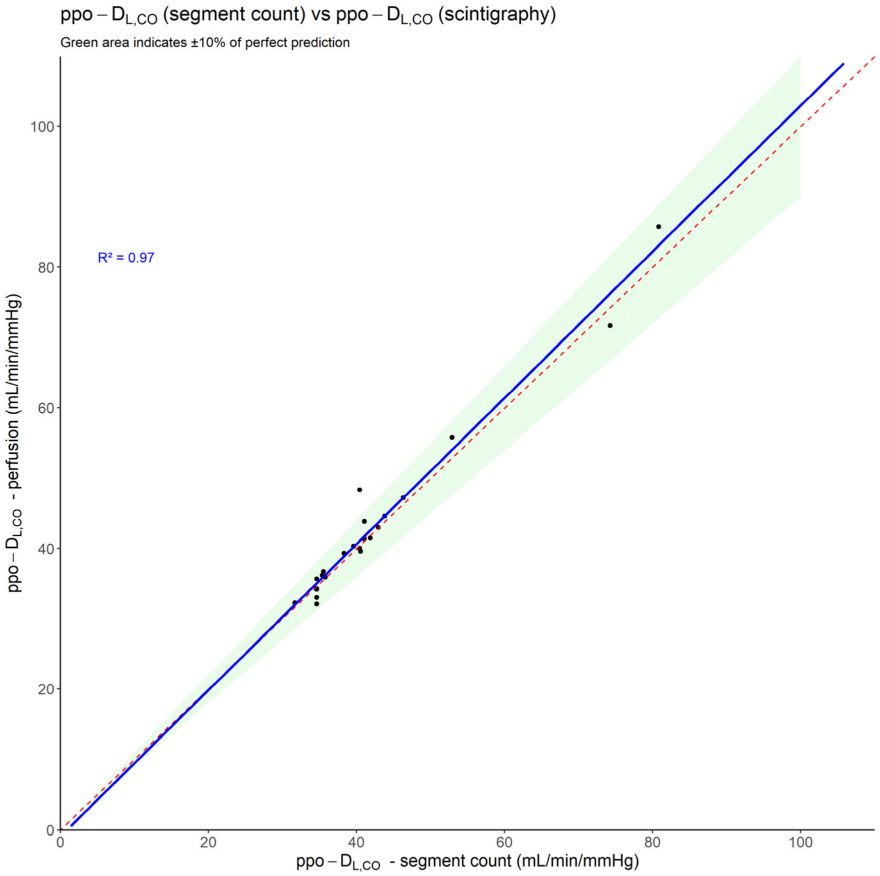

Figure S3. ppo-$D_{L,CO}$ (segment count) vs ppo-$D_{L,CO}$ (perfusion).

Linear regression of predicted postoperative diffusing capacity for carbon monoxide using the segment-counting method (x-axis) and perfusion (y-axis) for lobectomies and bilobectomies where perfusion scans where performed (n=23). Red dotted line indicates perfect relationship, green ribbon indicated ± 10 % of perfect relationship and black dots indicate each participant. Abbreviations: ppo: predicted postoperative; $D_{L,CO}$: diffusing capacity for carbon monoxide.
